# Analysis of BENCHMRK 1 & 2 using PhenoSense® assay for darunavir (DRV/r) resistance and exploration of functional monotherapy with RAL vs DRV

**DOI:** 10.1186/1758-2652-13-S4-P130

**Published:** 2010-11-08

**Authors:** J Rockstroh, J Eron, D Cooper, R Steigbigel, BY Nguyen, X Xu, H Wan, A Rodgers, M Miller, R Leavitt, P Sklar, H Teppler

**Affiliations:** 1 Universitat Bonn, Bonn-Venusberg, Germany; 2 University of North Carolina, Chapel Hill, USA; 3 Univeristy of New South Wales, Sydney, Australia; 4 Stony Brook University, Stony Brook, USA; 5 Merck, North Wales, USA

## Purpose of the study

Previous analyses of the 2 BENCHMRK studies of raltegravir (RAL) vs placebo (Pbo) plus optimized background therapy (OBT) in treatment-experienced HIV-infected patients (pts) by PSS as contributed by OBT used assumptions of susceptibility to DRV/r based on prior use, since commercial phenotyping was not available. Re-analysis is now performed using newly available DRV/r phenotype data.

## Methods

In BENCHMRK pts who used DRV/r in OBT, baseline PSS was recalculated using the DRV/r PhenoSense® result (Monogram Bioscience). A new analysis by PSS score of RNA <50c/mL for wk 48 and wk 156 was performed using the upper clinical cutoff (UC) of OBTs, including DRV/r. An exploratory analysis compared outcomes for pts whose only fully active ART was RAL or DRV/r.

## Results

184 pts in the RAL group and 99 in Pbo group used DRV/r in OBT at study entry; of these 166 and 90 pts, respectively, had no prior use of DRV/r and were previously considered DRV/r susceptible. 165 pts in the RAL group and 91 in Pbo group had baseline DRV/r Phenosense results: 7% and 7% of pts previously assumed susceptible to DRV/r showed phenotypic resistance; 17% and 44% assumed resistant to DRV/r were found to be susceptible.

Overall results at wk 48 were 64% vs 34% with RNA<50c/mL for RAL vs Pbo. Wk 48 virologic outcomes by PSS score are shown in table 1. Wk 156 outcomes by PSS were consistent (not shown). In the exploratory analysis comparing functional monotherapy with RAL (PSS=0) vs DRV/r (Pbo, PSS=1) at wk 48: 52% vs 30% of pts had vRNA <50 c/mL. Wk 156 results (not shown) were consistent with wk 48.

**Table 1:** 

	Efficacy at week 48, RNA<50 copies/mL			
	%, (n/N)			

	Initial approach		New analysis	

	(DRV/r phenotype assumed)		(DRV/r phenotype data)	

PSS	RAL	Placebo	RAL	Placebo

0	51 (17/33)	8 (1/12)	52 (16/31)	8 (1/13)

1	48 (34/71)	13 (7/54)	45 (34/79)	15 (8/55)

2	67 (107/160)	30 (26/88)	69 (102/148)	29 (24/83)

≥3	73 (112/153)	60 (39/65)	72 (113/156)	59 (38/64)

## Conclusions

In BENCHMRK, prior use of DRV predicted DRV susceptibility similarly to the UC phenotypic criteria. Re-analysis of virologic responses by PSS score incorporating the UC Phenosense result for DRV/r demonstrated consistent treatment differences between RAL and Pbo groups for all PSS scores, generally similar to the earlier analyses. In an exploratory analysis approximating a direct comparison of RAL vs DRV/r as sole active agents, virologic responses using UC appeared higher for RAL than DRV at both time points, although numbers of pts receiving DRV monotherapy were small.

